# Basal forebrain motivational salience signal enhances cortical processing and decision speed

**DOI:** 10.3389/fnbeh.2015.00277

**Published:** 2015-10-12

**Authors:** Sylvina M. Raver, Shih-Chieh Lin

**Affiliations:** Neural Circuits and Cognition Unit, Laboratory of Behavioral Neuroscience, National Institute on Aging, National Institutes of HealthBaltimore, MD, USA

**Keywords:** nucleus basalis, behavioral flexibility, attention, decision making, rat, gain modulation

## Abstract

The basal forebrain (BF) contains major projections to the cerebral cortex, and plays a well-documented role in arousal, attention, decision-making, and in modulating cortical activity. BF neuronal degeneration is an early event in Alzheimer’s disease (AD) and dementias, and occurs in normal cognitive aging. While the BF is best known for its population of cortically projecting cholinergic neurons, the region is anatomically and neurochemically diverse, and also contains prominent populations of non-cholinergic projection neurons. In recent years, increasing attention has been dedicated to these non-cholinergic BF neurons in order to better understand how non-cholinergic BF circuits control cortical processing and behavioral performance. In this review, we focus on a unique population of putative non-cholinergic BF neurons that encodes the motivational salience of stimuli with a robust ensemble bursting response. We review recent studies that describe the specific physiological and functional characteristics of these BF salience-encoding neurons in behaving animals. These studies support the unifying hypothesis whereby BF salience-encoding neurons act as a gain modulation mechanism of the decision-making process to enhance cortical processing of behaviorally relevant stimuli, and thereby facilitate faster and more precise behavioral responses. This function of BF salience-encoding neurons represents a critical component in determining which incoming stimuli warrant an animal’s attention, and is therefore a fundamental and early requirement of behavioral flexibility.

## Introduction

The mammalian basal forebrain (BF) is one of the most prominent cortically projecting neuromodulatory systems, with dense projections throughout the entire cerebral cortex, including prefrontal cortical areas (Gritti et al., 1997; Henny and Jones, 2008; Zaborszky et al., 2015). BF is an important structure implicated in attention, arousal, and in the control of cortical activity and plasticity (Everitt and Robbins, 1997; Wenk, 1997; Kilgard and Merzenich, 1998; Weinberger, 2003; Froemke et al., 2007). BF neuronal degeneration often occurs as an early event in Alzheimer’s disease (AD; Whitehouse et al., 1982; Grothe et al., 2012) and some forms of dementia (Cummings and Benson, 1984; Grothe et al., 2012). BF impairment has been implicated in normal cognitive aging (Gallagher and Colombo, 1995). In recent years, deep brain stimulation of BF targets has emerged as a potential novel therapy to alleviate dementia-related cognitive impairments (Freund et al., 2009; Hescham et al., 2013; Salma et al., 2014). Because of BF’s important role in normal cognitive functioning and in age-related diseases, understanding BF circuitry is therefore an important topic in neuroscience.

Despite the historical focus of BF studies on its cholinergic neurons, recent studies have begun to reveal the heterogeneity of neuronal dynamics and the functional significance of different non-cholinergic elements in the BF (a brief review in Lin et al., 2015). In this review, we focus on a specific population of putative non-cholinergic neurons in the BF that have been extensively studied in recent years (Lin et al., 2006; Lin and Nicolelis, 2008; Avila and Lin, 2014a,b; Nguyen and Lin, 2014). These studies highlight the functional significance of this group of putative non-cholinergic BF neurons in the decision making process via the encoding of motivational salience, which supports a fundamental aspect of behavioral flexibility.

In the first part of this article (Section 1), we discuss how the anatomical and neurochemical complexity of the BF extends far beyond the cholinergic neurons that have historically been the focus of study. In Section 2, we review recent studies that identify a unique population of putative non-cholinergic BF neurons that encodes the motivational salience of stimuli with a robust bursting response and discuss their neurochemical identity. In Section 3, we review previous BF single unit studies in behaving animals and suggest that this group of salience-encoding BF neurons have been widely described but interpreted under different circuit identities. In Section 4, we review the key features of salience-encoding BF neurons that have been revealed by recent studies. Finally, in Section 5, we propose a unifying hypothesis about the functional significance and neurochemical identity of BF salience-encoding neurons. We propose that these salience-encoding BF neurons serve as a gain-modulation mechanism to augment cortical processing of behaviorally relevant stimuli, and to modulate the speed of the decision process that enables flexible and adaptive behavior.

## Section 1: BF is a Neurochemically and Anatomically Complex Region

**Figure 1:**
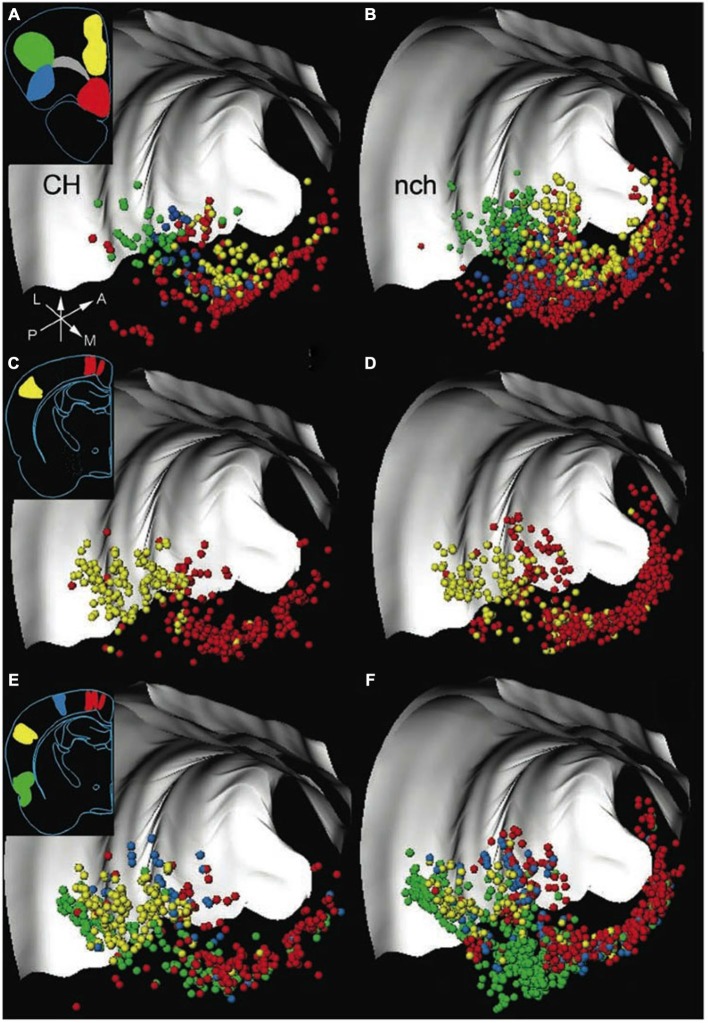
**Both cholinergic and non-cholinergic BF cortically projecting neurons are co-distributed across broad regions.** 3D distribution of neurons in the rat basal forebrain (BF), labeled by retrograde tracer injections into frontal and posterior cortical areas, with each row representing one experiment. The left column shows cortically projecting cholinergic (CH) neurons only; the right panel shows the distribution of non-cholinergic (nch) cortically projecting neurons. Insets show the locations of retrograde tracer injections in frontal and posterior cortical locations. Each cortical target, marked by a different color, receives projections (in corresponding colors to injection locations) from BF neurons distributed along a considerable rostro-caudal and dorso-ventral extent. Note that non-cholinergic projection neurons outnumber cholinergic neurons, and both cholinergic and non-cholinergic projection neurons are intermingled throughout the entire extent of the BF. Light gray structures are the corpus callosum and external capsule. Arrows show orientation (A, anterior; L, lateral; M, medial; P, posterior). Adapted from Zaborszky et al. (2015), reprinted with permission.

BF has traditionally been defined by the presence of cortically projecting magnocellular cholinergic neurons that provide most of the cholinergic input to the cerebral cortex (Meynert, 1872; Mesulam et al., 1983). The cortically-projecting cholinergic neurons do not reside in a single well-defined nucleus, but rather are distributed throughout a collection of brain regions that extend along both the anterior-posterior and dorso-ventral axes with a complex geometry (Figure 1; Gritti et al., 1997; Zaborszky et al., 2015). The regions containing cholinergic neurons can be broadly divided into two major divisions: an anterior division projecting to the hippocampus, that includes the medial septum and vertical band of Broca, and a posterior division projecting to the cerebral cortex and amygdala, that includes the substantia innominata (SI), the horizontal diagonal band of Broca (HDB), the magnocellular preoptic area (MCPO), and the nucleus basalis of Meynert (NBM; Meynert, 1872; Mesulam et al., 1983; Gritti et al., 2006; Zaborszky et al., 2015). Cortically-projecting neurons in the posterior BF division are also found throughout the posterior ventral pallidum (VP; Gritti et al., 2006; Zaborszky et al., 2015). The anterior division is commonly referred to as the medial septum, while the posterior division is commonly referred to as the BF. The current review focuses on the posterior division only and adopts this narrower definition of the term BF.

Despite the historical focus of BF studies on its cholinergic neurons, neuroanatomical studies in the last two decades have made it clear that BF contains more than just cholinergic neurons and is instead a neurochemically heterogeneous region. In addition to cholinergic neurons, the BF contains an equally prominent number of GABAergic and glutamatergic cortically projecting neurons that are spatially intermixed with cholinergic neurons and co-distributed throughout the BF (Figure 1; Freund and Gulyás, 1991; Freund and Meskenaitet, 1992; Gritti et al., 1997; Hur and Zaborszky, 2005; Henny and Jones, 2008; Zaborszky et al., 2015). While non-cholinergic BF neurons have historically been overlooked in the literature, their potential functional significance has been suspected in BF lesion studies: cholinergic-specific lesions of the BF produces limited behavioral and cognitive impairments, and does not capture the scope and severity of non-selective BF lesions that affect non-cholinergic neurons (Dunnett et al., 1991; Page et al., 1991; Muir et al., 1993; Wenk et al., 1994; Berntson et al., 2002). The functional significance of non-cholinergic BF neurons has received increasing attention in recent years (Sarter and Bruno, 2002; Lin and Nicolelis, 2008; Avila and Lin, 2014a; Nguyen and Lin, 2014; Kim et al., 2015) as studies have begun to reveal the heterogeneity of neuronal dynamics and the functional significance of different non-cholinergic elements in the BF (a brief review in Lin et al., 2015). The neurochemical heterogeneity in BF highlights the importance of identifying and characterizing the distinct component populations of BF circuits, especially in distinguishing the contribution of cholinergic neurons from non-cholinergic BF neurons.

The complex geometry of the BF also intersects at different subregions with several other macrosystems, such as the ventral-striatopallidal system and the extended amygdala, that have input-output connectivity patterns distinct from that of the BF (Gritti et al., 1997; Heimer, 2000). The spatial overlap with other macrosystems, as well as the anatomical heterogeneity between different sub-regions of the BF, add additional layers of complexity to the study of BF, and can become sources of confusion. It is therefore essential for studies to report the exact locations of their experimental investigations within the large BF complex, so that the functional contributions of BF can be distinguished from those of overlapping macrosystems.

## Section 2: BF Bursting Neurons Represent a Unique Population of Putative Non-Cholinergic BF Neurons

**Figure 2:**
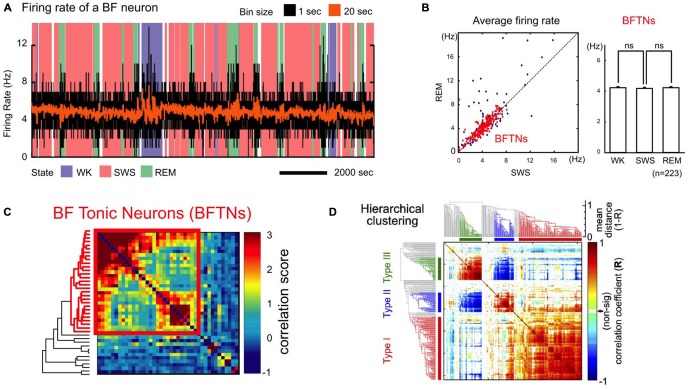
**A unique population of non-cholinergic BF neurons. (A)** Example firing rate trace of a BF bursting neuron overlaid on wake-sleep coding for the different arousal states [wake (WK); slow-wave sleep (SWS); REM sleep (REM)] and binned at 1 s (black) or 20 s (orange). **(B)** Average firing rates of BF bursting neurons. Left, BF tonic neurons (BFTNs, red), later identified to be BF bursting neurons or salience-encoding neurons (Lin and Nicolelis, 2008), have similar firing rates (generally < 10 Hz) during REM and SWS. Right, the average firing rates for BFTNs are not significantly different between the different arousal states. BF bursting neurons are therefore unlikely to be cholinergic neurons or PV + GABAergic neurons. **(C)** Pairwise correlation of spontaneous BF neuronal activity across arousal states was sorted using a hierarchical clustering algorithm with accompanying dendrogram aligned on the side (BFTNs in red). The activity of BFTNs are highly correlated with each other (bounded by red box), and minimally correlated with other non-BFTNs. **(D)** Pairwise correlation of BF neuronal responses to key behavioral events in a reward-biased reaction time (RT) task using a hierarchical clustering algorithm. The majority of recorded BF neurons show highly homogeneous response profiles that correspond to salience-encoding BF neurons (Type I), and which are distinct from two other neuronal populations in this region whose activities are locked to movement (Type II, III). **(A–C)** were adapted from Lin et al. (2006) reprinted here with permission. **(D)** was originally published in Avila and Lin (2014b).

Recent studies have identified a unique population of BF neurons that forms a physiologically and functionally homogenous ensemble, and that has been referred to as BF bursting neurons or salience-encoding BF neurons in the literature (Lin et al., 2006; Lin and Nicolelis, 2008; Avila and Lin, 2014a,b; Nguyen and Lin, 2014). The BF bursting neurons are characterized by three defining features: first, these neurons have low tonic firing rates (1–10 Hz) that remain unchanged across the different phases of the sleep-wake cycle (Figures 2A,B; Lin et al., 2006; Lin and Nicolelis, 2008). Second, the activities of these neurons are highly correlated with each other, and are punctuated by phasic ensemble bursting events that involve most BF bursting neurons (Figure 2C; Lin et al., 2006; Lin and Nicolelis, 2008). Third, these neurons show highly similar phasic bursting responses to motivationally salient stimuli that are distinct from other recorded neurons in this region (Figure 2D; Avila and Lin, 2014b; more discussion in the next section). The large amplitude action potentials with broad and complex waveforms (Avila and Lin, 2014b) of BF bursting neurons are consistent with the properties of large, magnocellular cortically projecting neurons previously described in the BF (Gritti et al., 1993, 1997). Furthermore, the short latencies in modulating cortical activity by BF bursting neurons (Nguyen and Lin, 2014) are consistent with the conduction delays of a direct BF projection to the cerebral cortex (Aston-Jones et al., 1985; Reiner et al., 1987). BF bursting neurons thus form a functionally and physiologically homogeneous population, most likely as a component of the BF corticopetal projection network. Recordings in the MS region do not find similar bursting neurons (Zhang et al., 2011), suggesting that neurons in the MS and BF regions do not share the same properties.

Multiple lines of indirect evidence suggest that BF bursting neurons do not match the known properties of BF cholinergic neurons. First, the constant firing rates in BF bursting neurons across different arousal states (Figures 2A,B) stands in contrast to BF cholinergic neurons whose firing rates are significantly higher during waking and REM sleep compared to slow-wave sleep (SWS; Lee et al., 2005; Hangya et al., 2015). Second, the instantaneous firing rates of BF bursting neurons within the bursts rarely exceed 80 Hz (Lin et al., 2006; Lin and Nicolelis, 2008), which is significantly slower than cholinergic BF neurons that can fire calcium bursts with much faster intra-burst frequencies (100–200 Hz or higher; Alonso et al., 1996; Lee et al., 2005; Hangya et al., 2015). Third, the temporal dynamics of BF bursting neurons in response to primary reinforcers do not match those of optogenetically identified BF cholinergic cells. A recent report (Hangya et al., 2015) reveals that cholinergic neurons can be precisely activated by primary reinforcers with very short latencies (15–40 ms), which is markedly faster than the BF bursting response to primary reinforcers that takes place between 50–200 ms after reinforcer delivery (Lin and Nicolelis, 2008; Avila and Lin, 2014a). These lines of evidence suggest that BF bursting neurons likely represent a unique group of non-cholinergic BF neurons.

In addition to the corticopetal cholinergic neurons, BF contains prominent populations of GABAergic and glutamatergic cortically projecting cells (Gritti et al., 1997; Henny and Jones, 2008; Zaborszky et al., 2015) that are likely candidates for the identity of the BF bursting neurons. The GABAergic BF neurons present an intriguing possibility because BF GABAergic projections to the cortex are ideally positioned to enhance cortical activity due to their preferential innervation of intracortical interneurons (Freund and Gulyás, 1991; Freund and Meskenaitet, 1992; Henny and Jones, 2008). While many cortically projecting GABAergic BF neurons also express the calcium binding protein parvalbumin (PV; Gritti et al., 1997), it appears unlikely that the BF bursting neurons correspond to the BF cortically projecting PV + GABAergic neurons. A recent study demonstrated that optogenetically tagged PV + GABAergic BF neurons have sustained firing rates greater than 30 Hz (Kim et al., 2015) and brief action potentials (McKenna et al., 2013), which are at odds with the low tonic activity (1–10 Hz) and broad action potential waveforms of BF bursting neurons. Furthermore, the firing rates of these PV + GABAergic BF projection neurons differ across the different sleep cycles, with activity between 25–50 Hz in wake and REM sleep that drops to less than 25 Hz during slow wave sleep (Kim et al., 2015), and further differentiates the activity of these neurons from the BF bursting neurons whose firing rates are not modulated by arousal states (Figures 2A,B; Lin et al., 2006; Lin and Nicolelis, 2008). Besides PV + GABAergic neurons, other populations of GABAergic projection neurons exist in BF and can be identified by their expression of the potassium channel Kv2.2 (Hermanstyne et al., 2010) or the neurokinin-3 receptor (Furuta et al., 2004). Another possibility is that BF bursting neurons represent direct glutamatergic BF projections to the cortex (Hur and Zaborszky, 2005). Together, the studies reviewed here suggest that BF bursting neurons are unlikely cholinergic or PV + GABAergic BF projection neurons, and suggest that they represent another group of non-cholinergic BF corticopetal neurons whose neurochemical identity remains to be defined.

## Section 3: Differing Interpretations of BF Salience-Encoding Neurons in the Literature

**Figure 3:**
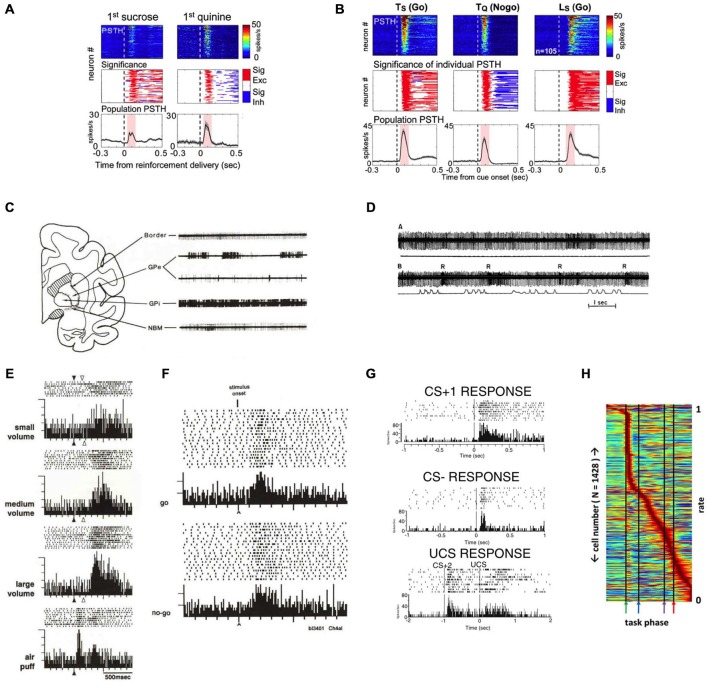
**Salience-encoding BF neurons have been widely reported in both non-human primates and rodents. (A)** Rodent salience-encoding BF neurons show robust bursting responses to both primary reward (sucrose) and punishment (quinine). Top panels, each row represents the peri-stimulus time histogram (PSTH) of one neuron. Middle panels indicate the presence of significant excitatory (red) or inhibitory (blue) responses. Bottom panels show population PSTHs of all salience-encoding BF neurons. **(B)** Rodent salience-encoding neurons show robust bursting responses to motivationally salient cues that predict rewards (Ts and Ls) and punishment (Tq) when animals made correct behavioral responses (Go vs. Nogo). Ts = tone predicting sucrose; Tq = tone predicting quinine; Ls = light predicting sucrose. Conventions are the same as in **(A)**. **(C)** The characteristic firing patterns differ between rhesus monkey BF structures (Border and NBM) and the globus pallidus (GP). Example 3.5 s traces from these regions reveal that neurons recorded in the nucleus basilis of Meynert (NBM) and the medullary lamina (border) have steady, regular tonic discharge patterns, in contrast to neurons in neighboring GP segments (external, GPe, and internal, GPi). **(D)** An example rhesus monkey BF neuron in the border region whose activity is not modulated by movement, but responds with bursts of action potentials to rewards. Action potential traces (top) are aligned with movement traces (bottom), with the first segment during rest (A; flat movement trace) and the second segment during push-pull arm movements (B; movement indicated in the bottom trace). Note how the border unit does not respond to the arm movements, but does burst to each presentation of a juice reward (R). **(E)** An example rhesus monkey NBM neuron responds with graded bursting responses to rewards, as well as to an aversive stimulus (air puff). **(F)** An example rhesus monkey NBM neuron shows robust bursting responses to the onset of stimuli instructing either a go response or a nogo response in order to receive reward. **(G)** An example neuron recorded in the rat caudal VP region shows robust bursting responses to a tone that predicts a sucrose pellet and instructs a go response (top; CS+1), a different tone that predicts no reward and instructs a nogo response (middle; CS−), and to the sound of the feeder that delivers a sucrose pellet (bottom; CS+2) as well as to the delivery of the pellet reward itself (UCS). **(H)** Normalized firing rates of all BF neurons recorded in a behavioral task, each neuron (*y*-axis) normalized to its maximum response across all phases of the task (*x* axis). While different sub-populations of BF neurons respond to all phases of a behavioral task, there is a clear overrepresentation of neurons that respond rapidly and robustly to the reward-predictive stimulus (first black line, green arrow). **(A,B)** were adapted from Lin and Nicolelis (2008); **(C–F)** were adapted from Richardson and DeLong (1991); **(D)** was adapted from DeLong (1971); **(G)** was adapted from Tindell et al. (2005), all reprinted here with permission. **(H)** was adapted from Tingley et al. (2014).

Perhaps the most distinct and best-characterized property of BF bursting neurons is their ability to encode the motivational salience of primary reinforcers and reinforcer-predictive cues using phasic bursting responses. In the rodent BF, Lin and colleagues have demonstrated that BF bursting neurons respond to both primary reward (water or a sucrose solution; Figure 3A; Lin and Nicolelis, 2008; Avila and Lin, 2014a,b; Nguyen and Lin, 2014) and punishment (a quinine solution; Lin and Nicolelis, 2008). As an animal learns the associative relationship between the reinforcers and the preceding conditioned stimuli (CS), both the CSs that predict reward (CS+) or punishment (CS−) acquire the ability to elicit robust bursting in BF neurons (Figure 3B). Given that the phasic bursting response is similarly elicited by the CS, irrespective of its sensory modality (auditory or visual), associated motor response (Go or Nogo), or hedonic valence (reward or punishment), the bursting response likely encodes the motivational salience of the stimulus (Lin and Nicolelis, 2008).

The phasic bursting responses of BF neurons to motivationally salient stimuli have in fact been widely described in both non-human primate and in rodent BF literatures. In non-human primates, DeLong first described in 1971 (DeLong, 1971) neurons in the primate SI/NBM region that fire with different response patterns and at tonically lower rates than the neighboring neurons in the globus pallidus (GP; Figure 3C), and that show bursting responses to the presentation of a juice reward (Figure 3D; Richardson and DeLong, 1991). These reinforcement-active neurons not only show graded response amplitudes according to reward amount (Richardson and DeLong, 1991), but also robustly burst to aversive stimuli, such as air puffs (Figure 3E; Richardson and DeLong, 1991). SI/NBM neurons were subsequently found to respond to the sensory cues that predict rewards, in addition to the primary reinforcers themselves (Figure 3F). An example is seen in Figure 3F that shows bursting activity of a primate NBM neuron to reward-predicting stimuli, regardless of whether the cue instructs the animal to make a movement (Go) or refrain from making a movement (Nogo) in order to obtain reward (Richardson and DeLong, 1991). Neurons distributed throughout the SI, NBM, and HDB nuclei of the BF therefore appear to reflect the reinforcing nature of rewards and their predictive stimuli (Wilson and Rolls, 1990). Subsequent studies confirmed that reward-related NBM neurons do not encode the sensory qualities of the reward-predicting cues (Wilson and Rolls, 1990; Richardson and DeLong, 1991).

More recent studies in the rodent BF have identified similar response patterns as the non-human primate BF bursting neurons (Tindell et al., 2005, 2009; Lin and Nicolelis, 2008; Smith et al., 2011; Tingley et al., 2014). Figure 3G shows examples of such neurons from the Aldridge group that respond with phasic bursting responses to conditioned stimuli that are associated with reward and Go responses (CS+) or with no reward and Nogo responses (CS−). Rodent BF neurons also respond to primary reinforcers with similar responses regardless of whether animals receive appetitive outcomes, such as a sucrose solution or pellet (Tindell et al., 2005; Lin and Nicolelis, 2008), or an aversive outcome like a hypertonic salt solution or quinine (Tindell et al., 2005, 2009; Lin and Nicolelis, 2008; Smith et al., 2011). Similarly, Figure 3H shows the entire neuronal population recorded in the BF region from the Nitz group (Tingley et al., 2014), and shows an overrepresentation of neurons with phasic bursting responses to CS onset.

It is important to note that salience-encoding BF neurons are also influenced by hedonic valence. For example, subsequent to the initial phasic bursting response to both CS+ and CS− in a Go/Nogo task that encodes motivational salience, Lin and Nicolelis (2008) showed that the initial bursting is followed by a sustained phase of activity modulation that is excitatory in rewarded (Go) trials and inhibitory in punishment (Nogo) trials (Figure 3B). Sustained responses of BF bursting neurons reflecting the hedonic valence of the predicted outcome are also reported in other studies (Wilson and Rolls, 1990; Richardson and DeLong, 1991; Tindell et al., 2006, 2009) and appear to track the updated value of the expected outcome (Tindell et al., 2009; Smith et al., 2011). Future studies will need to address how motivational salience and hedonic valence information coexist in the same neuronal population.

The prevalence of salience-encoding neurons in the BF literature shows that this is a prominent neuronal population widely present in both rodents and non-human primates. Despite their prevalence, BF salience-encoding neurons have often been interpreted very differently in the literature as either the BF cholinergic neurons (Wilson and Rolls, 1990; Richardson and DeLong, 1991; Tingley et al., 2014), or as corresponding to ventral pallidal (VP) neurons as part of the ventral striatopallidal system (Tindell et al., 2005, 2009; Smith et al., 2011). As described in Section 2, multiple physiological and functional features of these salience-encoding neurons differ from those of cholinergic BF neurons, including their bursting characteristics, their lack of modulation by sleep-wake states (Figures 2A,B), and their response latencies to reinforcers (Lin et al., 2006; Lin and Nicolelis, 2008; Hangya et al., 2015). On the other hand, although the location of BF salience neurons overlaps with the caudal VP, bursting neurons have been found both above and below the caudal VP region, broadly corresponding to regions that contain cortically-projecting BF neurons (Lin et al., 2006; Lin and Nicolelis, 2008; Avila and Lin, 2014a,b; Nguyen and Lin, 2014). Moreover, unlike other neurons in this region that encode movement and better resemble neurons in the striatopallidal circuit, salience-encoding BF neurons are concerned primarily about motivationally salient events but not movement (Figure 2D; Avila and Lin, 2014b).

In this context, the unique contributions of Lin and colleagues are the identification of salience-encoding neurons as a physiologically and functionally homogeneous neuronal population in the BF, which highlights the importance in distinguishing BF salience-encoding neurons from the other neurons in this region. More importantly, Lin and colleagues suggest that these neurons are non-cholinergic BF neurons that project to the cerebral cortex (Lin et al., 2006; Lin and Nicolelis, 2008; Avila and Lin, 2014b), which stands in stark contrast with previous interpretations that attribute this phenotype to either cholinergic BF neurons or to VP neurons. These differing interpretations underscore the anatomical and neurochemical heterogeneity of the BF, as salience-encoding neurons represent but one functionally and physiologically homogenous population among many others that respond to different behavioral events and play key roles in value-laden decisions. These differing accounts also underscore the importance in future studies to determine the neurochemical identity, as well as the projection targets, of salience-encoding BF neurons.

## Section 4: Key Features of the BF Salience-Encoding Neurons in the Decision-Making Process

In this section, we highlight several key features of BF bursting neurons and describe how BF bursting activity quantitatively modulates behavioral responses and cortical processing. These features are instrumental in understanding the functional significance of BF bursting neurons in the decision-making process.

**Figure 4:**
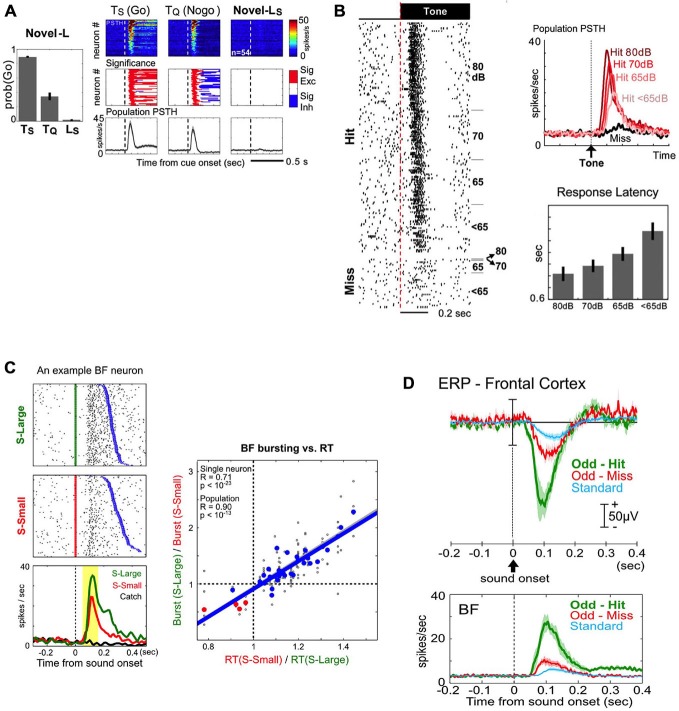
**Key features of BF salience-encoding bursting neurons in the decision-making process. (A)** BF bursting responses to motivationally salient cues are acquired through associative learning. Left: behavioral responses to auditory cues that rats have learned to associate with sucrose (Ts) and quinine (Tq), and the lack of behavioral response to a novel light cue that would subsequently come to predict sucrose (Ls). Right: BF bursting responses are present only for the previously learned cues (Ts and Tq), but not for the perceptually salient, but motivationally not salient, novel Ls cue. Conventions for this figure are the same as in Figure 3A. **(B)** BF bursting response is tightly coupled with successful behavioral response to motivationally salient cues. Left: all-or-none bursting responses of an example BF neuron to tone onsets in successful (Hit) or unsuccessful (Miss) trials in a near-threshold auditory detection task, with trials sorted by sound intensity levels (dB). BF bursting responses are always present in Hit trials, regardless of the sound intensity of the stimulus. Top right: population PSTHs for BF neurons to tones for Hit (shades of red) or Miss (black) trials. Note that, although the bursting response is present in all Hit trials, the amplitude of the burst is graded based on the detectability of the tone, and associated with overall response latencies (right bottom). **(C)** BF bursting amplitude is coupled with decision speed. Left: bursting responses of an example BF neuron to stimuli predicting a large (S-Large, green) or small reward (S-Small, red), sorted by reaction time (RT, blue). Note the larger amplitude bursting responses and faster RTs to S-Large vs. S-Small stimuli. Right: significant correlation between BF bursting amplitude modulation and RT modulation, each calculated as a ratio between S-Large and S-Small trials. **(D)** BF bursting responses to motivationally salient cues enhance cortical processing by generating a frontal cortex event related potential (ERP) in an auditory oddball task. Top panel: the amplitudes of the frontal cortex ERP during the oddball task are graded with motivational salience, as they are higher for motivationally salient tones (Odd-Hit and Odd-Miss) than for the standard tone that does not require a response Bottom panel: both the amplitude and timing of the BF busting response scale with the simultaneously recorded frontal ERP in the top panel. **(A,B)** are adapted from Lin and Nicolelis (2008), reprinted here with permission. **(C)** is adapted from Avila and Lin (2014a), and **(D)** is adapted form Nguyen and Lin (2014).

The first key property of BF salience-encoding neurons is that their bursting responses to sensory stimuli are not innate, but are instead acquired through associative learning (Lin and Nicolelis, 2008). As neutral sensory stimuli acquire motivational salience through associative learning, they become conditioned stimuli (CSs) that reliably predict reward or punishment and can robustly elicit behavioral responses; simultaneously, the CSs also acquire the ability to elicit phasic bursting responses. The BF bursting response, however, is absent following other clearly perceptible but not motivationally salient stimuli. Figure 4A provides an example of BF neurons that display phasic bursting responses to previously learned motivationally salient cues, but at the same time show no response to a perceptually salient house light that the animal has not yet learned to associate with reward. Furthermore, as the association between stimuli and their predictive outcomes is reversed through extinction training, BF bursting responses to cues quickly diminish as cues lose their motivational salience (Lin and Nicolelis, 2008). These response patterns indicate that the BF bursting response is not required for the perception of a sensory cue, and its influence on the decision making process must take place after the initial perception stage.

The second key property of BF salience-encoding neurons is that the bursting response is tightly coupled with the success of behavioral responses to motivationally salient cues. In a near-threshold auditory detection task, BF neurons displayed phasic bursting responses to tones when animals made correct behavioral responses (Hit; Figure 4B), even when tones were presented at or below detection level threshold. In contrast, when animals failed to respond to the tone, BF neurons were not activated (Miss; Figure 4B; Lin and Nicolelis, 2008). Furthermore, within trials in which animals successfully detected and responded to the tone, the amplitude of the BF bursting response scaled with the animals’ response latency (Figure 4B; Lin and Nicolelis, 2008). These results suggest that successful responses to the CS are associated with, and perhaps require, the BF motivational salience signal, which likely facilitates the execution of the correct behavioral response based on perceived cues. Consistent with this interpretation, in the Go/Nogo task, incorrect “false-alarm” responses in Nogo trials were associated with higher BF activity compared with correct Nogo responses (Lin and Nicolelis, 2008, Supplemental Figure S4).

The third key property of BF bursting neurons is that the strength of the BF motivational salience signal is quantitatively coupled with faster and more precise decision speeds. To determine the quantitative relationship between the BF salience signal and decision speed, Avila and Lin (2014a) investigated whether the BF bursting amplitude is capable of influencing the earliest read out of behavioral responses to the CS using the metric of simple reaction time (RT). In a reward-biased simple RT task, the motivational salience of two auditory cues was manipulated by the magnitude of associated rewards. The cue that predicted a large reward elicited faster RTs and stronger BF bursting amplitudes and importantly, the magnitude of RT modulation was quantitatively accounted for by the modulation of BF bursting amplitudes (Figure 4C; Avila and Lin, 2014a). The relationship between the BF bursting response and RT was found to be causal, as augmenting the strength of the BF bursting response with BF electrical simulation increased decision speed (Avila and Lin, 2014a). These findings suggest that the BF bursting response may serve as a gain modulation signal of the decision making process to enhance the speed of responding to motivationally salient cues.

The fourth key property is that the BF bursting response enhances cortical processing at least in part by generating an event-related potential (ERP) response in the frontal cortex (Figure 4D; Nguyen and Lin, 2014). To better understand how the BF motivational salience signal modulates downstream cortical processing, Nguyen and Lin (2014) studied the relationship between the BF bursting response and the ERP response in the frontal cortex using an auditory oddball task (Figure 4D). The amplitude and timing of BF bursting and the prominent frontal ERP response were tightly coupled with each other (Figure 4D), and such coupling was observed on a trial-by-trial basis (Nguyen and Lin, 2014). Furthermore, the frontal ERP response was associated with local field potential (LFP) responses localized to deep cortical layers of the frontal cortex, coincident with the target layers of BF projections (Henny and Jones, 2008). Such layer-specific LFP response patterns are also recreated by BF electrical stimulation with a delay of 5–10 ms (Nguyen and Lin, 2014), consistent with the conduction delay from the BF to the frontal cortex (Aston-Jones et al., 1985; Reiner et al., 1987). These observations suggest that the frontal ERP/LFP response likely represents the first step by which the BF motivational salience signal enhances cortical processing of a perceived stimulus to facilitate correct behavioral responses.

## Section 5: Hypothesis

**Figure 5:**
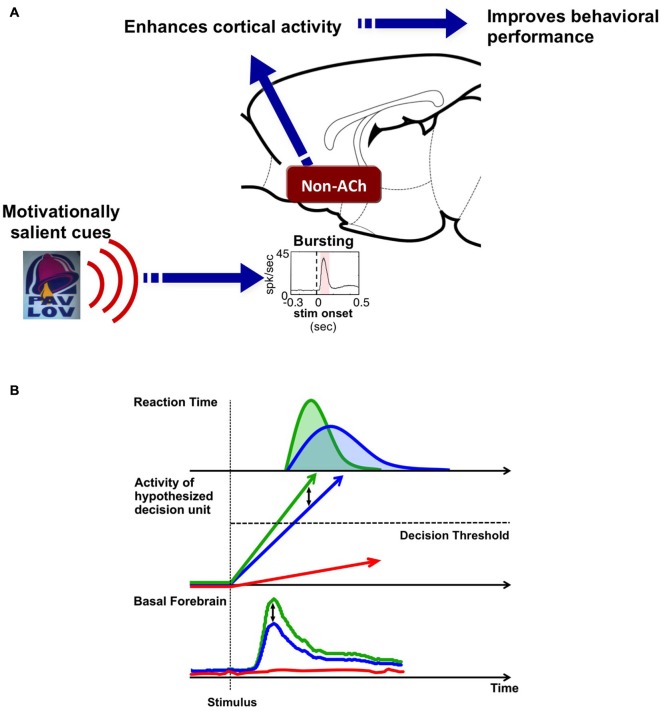
**Hypothesis: BF salience-encoding neurons act as a gain modulation signal to enhance cortical processing and the speed of decision-making. (A)** Our working hypothesis contains three key components: first, a unique population of non-cholinergic BF neurons encodes the motivational salience of stimuli using a phasic bursting response. Second, BF motivational salience is rapidly broadcasted to the cerebral cortex to enhance cortical processing. Third, this modulation results in faster and more precise decision speed. **(B)** The role of BF motivational salience signal in the decision making process. Simple decision-making is commonly modeled as a drift-diffusion process or a linear rise to threshold process where activity accumulates toward a decision threshold. We propose that the amplitude of the BF motivational salience signal serves as a gain modulation mechanism that controls the rate of activity accumulation in the decision unit. Stronger BF motivational salience signal (green) increases the rate of activity accumulation relative to weaker BF bursting (blue), and in turn, increases decision speed and generates a faster RT distribution. On the other hand, the absence of BF motivational salience signal (red) translates into the decision unit never reaching the decision threshold, and in turn, leads to no behavioral response (absence of red RT distribution).

Based on studies reviewed above, we propose a unifying hypothesis that the BF salience-encoding neurons serve as a signal amplifying, or gain-modulation, mechanism for motivationally salient cues (Figure 5A). The hypothesis includes three key components: (1) A unique population of putative non-cholinergic BF neurons encodes the motivational salience of stimuli with a phasic bursting response (Lin et al., 2006; Lin and Nicolelis, 2008); (2) The BF motivational salience signal is rapidly broadcasted to the cerebral cortex to enhance cortical processing (Nguyen and Lin, 2014); and (3) This modulation results in faster and more precise decision speed (Avila and Lin, 2014a).

This hypothesis addresses a fundamental question in neuroscience: how the brain filters meaningful from meaningless stimuli to execute responses only to stimuli that are behaviorally relevant. Animals are constantly faced with a barrage of incoming sensory stimuli; however, most of the stimuli are not motivationally salient, do not carry any behavioral consequence, and need not be responded to. For the subset of stimuli that are motivationally salient, which may or may not be perceptually salient, the brain must require an internal gain modulation mechanism to amplify their processing and ensure correct and efficient behavioral responses. Such is the main behavioral function of this unique population of non-cholinergic BF bursting neurons, to serve as a fast and powerful gain modulation mechanism to facilitate behavioral responses to environmental stimuli, and that operates based on the motivational, but not perceptual, salience of the stimuli.

This gain-modulation hypothesis can also be conceptualized in a decision model (Figure 5B). Simple decision making processes have been commonly modeled as activity accumulation in a hypothetical decision unit, such as the drift-diffusion model or the linear rise to threshold model (Ratcliff and Rouder, 1998; Reddi and Carpenter, 2000; Ratcliff, 2001). Once the activity of this decision unit reaches a threshold, a decision is made and a behavioral response, such as the RT response, is observed. The studies reviewed here suggest that BF bursting response serves as a gain modulation signal that modulates the rate of activity accumulation in the decision unit. A stronger BF bursting response—such as that generated in response to a stimulus with high motivational salience—increases the rate of activity accumulation, and in turn, increases decision speed and generates a faster RT distribution. Data collected by Lin and colleagues support this hypothesis (Figure 5B): stimuli with greater motivational salience produce stronger bursting responses in putative non-cholinergic BF neurons (Lin and Nicolelis, 2008), that in turn enhances activity within cortical networks (Lin et al., 2006; Nguyen and Lin, 2014), and increases the speed and precision of decision making (Avila and Lin, 2014a). On the other hand, the absence of BF bursting in the near-threshold auditory detection task is coupled with the absence of a behavioral response, and likely reflects a lack of internal amplification, such that activity in the decision unit never reaches the decision threshold (Lin and Nicolelis, 2008).

The specific cortical mechanisms that underlie the transference of the BF motivational salience signal into a rapid and precise behavioral response remain to be determined, and should be the focus of future experiments. However, the ability of BF bursting neurons to rapidly enhance cortical activity and decision speed are consistent with a disinhibition mechanism mediated by GABAergic BF cortically projecting neurons. Anatomical data show that corticopetal GABAergic neurons preferentially innervate inhibitory interneurons in the neocortex (Freund and Gulyás, 1991; Freund and Meskenaitet, 1992; Henny and Jones, 2008). As these cortical GABAergic interneurons in turn each contact multiple excitatory pyramidal neurons, inhibition of interneuron activity by BF corticopetal projections would have the net result of inducing potent and widespread cortical excitation. Indeed, this disinhibition mechanism has been previously suggested to account for the ability of the BF’s non-cholinergic population to gate cortical information processing (Dykes, 1997; Sarter and Bruno, 2002). Additional experiments that confirm the neurochemical identity of the BF salience neurons and their projection targets are needed to test this disinhibition hypothesis, as a direct glutamatergic BF projection to the cortex (Hur and Zaborszky, 2005) remains a possibility.

The BF’s ability to encode the motivational salience of a stimulus is a critical component in determining whether or not to attend to incoming sensory information, and is therefore a fundamental and early requirement of adaptive and flexible behavior. Indeed, animals can flexibly respond to the same stimulus depending on its associated motivational salience. The associated motivational salience can be dynamically adjusted through associative learning and rapidly reversed by extinction (Lin and Nicolelis, 2008). As such, the putative non-cholinergic BF salience-encoding neurons represent an important neural circuit that is instrumental in behavioral flexibility. Future experiments should be designed to test the specific contributions of the BF motivational salience signal in guiding flexible and adaptive behavior, and to provide a clearer understanding of the functions of this BF population in age-related diseases and normal cognitive aging.

## Funding

This work was supported by the Intramural Research Program of the National Institute on Aging, National Institutes of Health.

## Conflict of Interest Statement

The authors declare that the research was conducted in the absence of any commercial or financial relationships that could be construed as a potential conflict of interest.
